# Gelseminum Sempervirens

**Published:** 1857-08

**Authors:** 


*Gelseminum Sempervirens—Yellow Jessamine*—Dr. J. A. Mayes in a report to the South-Carolina Medical Association thus alludes in his conclusion to this remedy, "I regard it as a direct sedative; more safe and manageable than veratrum viride, and more generally applicable in practice. I esteem it a most valuable adjuvant to other treatment in all cases where high arterial action exists, in which it is desirable to lessen the frequency of the pulse and to calm excitement, and where, as in cases of injury, it is desirable to lessen the irritability of the nervous system." He describes it as producing, in over-doses, most powerful prostration of the muscular system. He says: "Dr. Batchelor thus describes its properties," "The Gelseminum possesses Narcotic, Nervine, anti-spasmodic, and sedative powers. In full doses, it produces a sort of intoxication, languor and dizziness, double vision, inability to raise the eye-lids; and in an over-dose complete muscular prostration."

